# Comparative Gene Expression Analysis of Epithelioid Pleural and Peritoneal Mesothelioma

**DOI:** 10.3390/cancers18183018

**Published:** 2026-09-17

**Authors:** Rebecca Sparavelli, Anello Marcello Poma, Pinuccia Faviana, Clara Ugolini, Marco Lucchi, Piero Vincenzo Lippolis, Greta Alì, Rossella Bruno

**Affiliations:** 1 Unit of Anatomic Pathology, Department of Surgical, Medical and Molecular Pathology and Critical Care Medicine, University of Pisa, 56126 Pisa, Italy; rebecca.sparavelli@phd.unipi.it (R.S.); marcello.poma@med.unipi.it (A.M.P.);; 2 General and Peritoneal Surgery Unit, Azienda Ospedaliero-Universitaria Pisana, 56124 Pisa, Italy; 3 Unit of Pathological Anatomy 3, University Hospital of Pisa, 56126 Pisa, Italy

**Keywords:** mesothelioma, pleural, peritoneal, gene expression, extracellular matrix, molecular profile, NanoString, tumor microenvironment

## Abstract

**Background/Objectives**: Pleural and peritoneal mesothelioma (PL and PE) are aggressive diseases, accounting for 68.0–92.4% and 6.9–24.1% of all cases. To improve both diagnosis and management of these patients, a deeper knowledge and characterization of the molecular background are necessary. Although molecular profiling of PL has been largely investigated in recent years, much less is known about the genomic landscape of PE, due to its rarity. Here, we aim to compare gene expression profiling of pleural and peritoneal mesothelioma to underline main differences and to identify new potential histotype-specific biomarkers. **Methods**: We performed a gene expression analysis of 32 epithelioid PL and 21 epithelioid PE cases, diagnosed from 2017 to 2023 at the University Hospital of Pisa, using an nCounter custom panel including 117 target genes known for their crucial role in cancer. **Results:** PE, compared to PL, showed different expression levels of genes related to extracellular matrix modification (*ECM*), as collagen alteration, pro-inflammatory and angiogenic response (mediated by NF-κB). In detail, in PE we observed a significant upregulation of metalloproteinase-1 (*MMP1*), E-selectin (*SELE*), pappalysin-1 (*PAPPA*), estrogen receptor-2 (*ESR2*) and transportin-2 (*TNPO2*); and a downregulation of pyruvate kinase (*PKM*). Furthermore, BAP1 loss was associated with distinct gene deregulation in PE and PL; the only exception was *ASS1* upregulation, which was observed in both histologies. These insights may help refine the utility of *BAP1* as a biomarker. **Conclusions**: Deregulated genes in PE deserve further investigation as potential biomarkers, according to their biological role. In fact, *MMPs* and *PAPPA* impact migration ability and angiogenic response of mesothelioma, while *PKM* and *ESR2* are usually investigated for their involvement in reprogramming of energy metabolism. The identification of histotype-specific biomarkers is warranted to improve mesothelioma management.

## Simple Summary

Pleural and peritoneal mesothelioma are aggressive tumors with different molecular features. In this study, we compared gene expression profiles of 32 epithelioid pleural and 21 epithelioid peritoneal mesotheliomas using an nCounter custom panel including 117 cancer-related genes. Pleural and peritoneal mesothelioma showed a partially overlapping gene expression profile, but also some distinct molecular features that may reflect differences in tumor biology. The main differences involved genes associated with extracellular matrix remodeling, inflammation, angiogenesis, and energy metabolism pathways. In particular, *MMP1*, *SELE*, *PAPPA*, *ESR2*, and *TNPO2* were upregulated, whereas *PKM* was downregulated in peritoneal mesothelioma in comparison to pleural mesothelioma. Moreover, BAP1 loss was associated with different gene expression patterns in the two histotypes, although *ASS1* was upregulated in both. These findings highlight potential relevant molecular differences between pleural and peritoneal mesothelioma and suggest that distinct biological processes may contribute to their clinical characteristics. The identification of differentially expressed genes and altered biological pathways may improve the molecular characterization of mesothelioma and support the identification of novel biomarkers.

## 1. Introduction

Mesothelioma is a rare and highly aggressive malignancy, linked to asbestos exposure, with a latency period of 30–40 years [1]. It arises from the mesothelial lining of serous cavities, including pleura, peritoneum, pericardium, and tunica vaginalis [2]. Pleural (PL) and peritoneal mesothelioma (PE) account for 70% and 15% of diagnoses, respectively [3]. The histological subtypes of both PL and PE include epithelioid, which is the most common, sarcomatoid and biphasic subtypes [4].

Although PL and PE share several features, some important differences are well recognized. PL is more common in males, whereas PE occurs with similar frequency in males and females. PE generally affects younger patients, and a substantial proportion of cases are considered idiopathic, with a weaker association with asbestos exposure [5].

Beyond their clinicopathological characteristics, the molecular differences between PL and PE have not been fully elucidated. Indeed, most available studies investigating the molecular landscape of mesothelioma have focused on PL, whereas considerably less is known about PE.

Only a few comparative genomic studies are available. Both PL and PE predominantly harbor inactivating mutations in tumor suppressor genes, including *BAP1, NF2*, and *CDKN2A*, and a substantial proportion of mesothelioma patients harbor tumors characterized by homologous recombination deficiency [2]. In addition, Hitbrunner et al. found that peritoneal, compared to pleural mesothelioma, is characterized by a lower prevalence of alterations in *CDKN2A*, *CDKN2B* and *MTAP* genes [6]. Interestingly, compared with PL, some PE cases may harbor gene rearrangements involving target genes like *ALK* [7]. However, the somatic mutation rate in mesothelioma is relatively low compared with that of other solid tumors. In this context, the analysis of gene expression profiles may provide a more comprehensive characterization of the tumor molecular landscape [8].

A recent meta-analysis identified multiple differentially expressed genes (DEGs) in pleural mesothelioma, compared to normal pleural tissue [9]. Functional enrichment analysis revealed the involvement of several biological pathways and processes, notably inflammation, angiogenesis, cell adhesion and migration, cellular responses to stimuli, and lipid metabolism [9]. Another study showed that, compared with normal pleura, mesothelioma exhibits an immunologically defensive and relatively isolated molecular phenotype, characterized by the downregulation of adhesion molecules, cytokine receptors and ligands, as well as genes involved in inflammatory responses [10].

Regarding PE, Varghese et al. reported the overexpression of genes involved in the mTOR–PI3K pathways, which was associated with shorter survival in patients with PE treated with surgical cytoreduction and regional intraoperative chemotherapy perfusion. Overall, most of the knowledge regarding PE has been extrapolated from studies of pleural mesothelioma, which has hampered the identification of novel prognostic and therapeutic biomarkers specific to PE.

Despite numerous studies investigating the biological mechanisms underlying these tumors [11,12], therapeutic options remain limited for both PL and PE. Until recently, platinum plus pemetrexed (PEM) chemotherapy has been the standard first-line treatment for unresectable PL. In 2020, a combination of nivolumab and ipilimumab was approved as a first-line immunotherapy option [13]. However, not all patients derive significant benefit from these treatment approaches. For PE, cytoreductive surgery combined with hyperthermic intraperitoneal chemotherapy remains the standard therapeutic approach [14,15].

Given the rarity of PE and the limited characterization of its molecular profile, a direct comparison with PL could fill an important knowledge gap and provide clinically relevant information on site-specific molecular alterations, ultimately facilitating the identification of novel site-specific biomarkers.

In this study, we directly compared the gene expression levels of 117 cancer genes between epithelioid PE and PL to reveal potential mesothelioma type-specific biomarkers. Moreover, we investigated the transcriptional impact of *BAP1* protein loss, a key diagnostic and prognostic marker currently used in mesothelioma [16].

## 2. Materials and Methods

### 2.1. Sample Selection

A retrospective consecutive series of 32 epithelioid PL and 21 epithelioid PE patients, diagnosed from 2017 to 2023 at the University Hospital of Pisa, were included in this study. Histological and cytological diagnoses were rendered by expert pathologists in accordance with the 2021 WHO histological and immunohistochemical criteria [17]. For histological assessment, the most representative formalin-fixed paraffin-embedded (FFPE) block from each case, containing tumor areas, was selected for analysis.

This study was conducted in accordance with the Declaration of Helsinki and its subsequent amendments. Written informed consent was obtained from all patients prior to tumor biopsy or surgical treatment. All data were fully anonymized, and no sensitive personal information was collected or analyzed. The study did not alter or interfere with standard clinical practice.

### 2.2. Nucleic Acid Extraction and Purification

For each sample, four unstained sections (5 µm thick) were deparaffinized with xylene and rehydrated through a graded ethanol series. Manual macrodissection was performed to enrich for tumor cells. Total RNA was isolated using the RNeasy FFPE Kit (Qiagen, Hilden, Germany), according to the manufacturer’s instructions, and eluted in 18 µL of RNase-free water. The RNA concentration and purity were assessed using a spectrophotometer (Xpose, Trinean, Gentbrugge, Belgium) [18].

### 2.3. Gene Expression Analysis

Given the rarity of PL and PE, retrospective archival FFPE samples were used. The highly degraded RNA obtained from these tissues precluded whole-transcriptome sequencing. nCounter technology, based on hybridization and color-barcoded digital count of signals, was preferred because of its robustness in analyzing poor-quality RNA and its ability to directly quantify mRNA molecules without reverse-transcription steps [19]. Gene expression analysis was performed using a custom panel comprising 117 genes (NanoString Technologies, Seattle, WA, USA). Target genes were initially selected based on their involvement in cancer-related pathways, including cell-cycle regulation, focal adhesion, proliferation, extracellular matrix remodeling, and signaling pathways, and were subsequently selected based on their previously reported deregulation in mesothelioma, particularly PL [18].

Briefly, 100 ng of total RNA from each sample was hybridized with the nCounter CodeSet. mRNA quantification, including sample preparation, hybridization, target detection, and data acquisition, was performed according to the manufacturer’s instructions. Data normalization was carried out following the standard nCounter workflow using nSolver v4.0 software (NanoString Technologies). Briefly, raw counts were first subjected to technical normalization using positive-control probe sets to correct for assay variability and subsequently to biological normalization using the reference genes included in the CodeSet. The normalized data were log2-transformed and used as input for downstream analyses. Unsupervised hierarchical clustering and principal component analysis (PCA) were performed on normalized gene expression data to visualize sample distribution in reduced-dimensional space and to assess whether PL and PE samples exhibited distinct transcriptional profiles.

### 2.4. BAP1 and Ki67 Immunohistochemistry

BAP1 immunohistochemistry (IHC) was performed on FFPE tissue sections using a BenchMark XT automated slide stainer (Ventana Medical Systems, Tucson, AZ, USA). Sections were incubated with a mouse monoclonal anti-BAP1 antibody (clone C-4; Santa Cruz Biotechnology, Dallas, TX, USA) at a 1:100 dilution. Signal visualization was achieved using the OptiView DAB IHC Detection Kit combined with the OptiView Amplification Kit (Ventana, Tucson, AZ, USA). BAP1 loss was defined as the complete absence of nuclear immunoreactivity in all atypical mesothelial cells, provided that non-neoplastic stromal cells exhibited positive internal nuclear staining.

Ki67 expression was evaluated by IHC using the CONFIRM anti-Ki67 (30-9) rabbit monoclonal primary antibody (Roche Diagnostics, Tucson, AZ, USA). Proliferation index was assessed by counting the percentage of Ki67-positive tumor cells. A minimum of 1000 tumor cells were counted in the areas of highest proliferative activity (‘hotspots’) under high-power fields (×400 magnification). Only cells with distinct nuclear staining were considered positive.

### 2.5. Data Analysis

Differences between categorical variables were assessed with Fisher’s exact test. Continuous variables were presented as median and interquartile range (IQR), and differences were tested with the Mann-Whitney U test.

Differential expression analysis was performed following the procedures of the limma R package and using moderated *t*-statistics. *p*-values were adjusted using the Benjamini–Hochberg correction, and a false discovery rate (FDR) below 0.1 was deemed significant. The results of differential expression analysis were presented in a volcano plot.

To investigate the transcriptional impact of BAP1 protein loss, an additional differential expression analysis was performed according to BAP1 IHC status (lost vs. retained), excluding samples with missing BAP1 IHC annotation. Given the biological differences between pleural and peritoneal mesothelioma, this analysis was carried out using a linear model including histology, BAP1 IHC status, and their interaction term. This approach allowed estimation of (i) the effect of BAP1 loss within pleural mesothelioma, (ii) the effect of BAP1 loss within peritoneal mesothelioma, and (iii) genes showing a significantly different BAP1-associated transcriptional pattern according to histologic site. Differential expression analysis was performed using the limma R package with moderated t-statistics. Because this analysis was considered hypothesis-generating and was conducted on a limited sample size, an exploratory FDR threshold of 0.25 was adopted to define significant genes. Results were visualized by volcano plots for each contrast, and PDCD1 mRNA expression was additionally represented by violin plots according to the combined histology/BAP1 IHC groups.

Exploratory correlations between the Ki67 proliferation index and gene expression were assessed in PE only. For each gene in the expression matrix, Spearman’s rank correlation coefficient (rho) with Ki67 was calculated, together with the corresponding nominal *p*-value. Multiple testing was controlled using the Benjamini–Hochberg FDR adjustment. All statistical analyses and plots were generated in the R environment (version 4.5.3; last accessed 4 September 2026).

## 3. Results

### 3.1. Case Series

A series of 32 PL and 21 PE were examined in accordance with the 2021 WHO histological and immunohistochemical criteria (Table 1).

### 3.2. Normalization and Unsupervised Analyses

**Figure 1:**
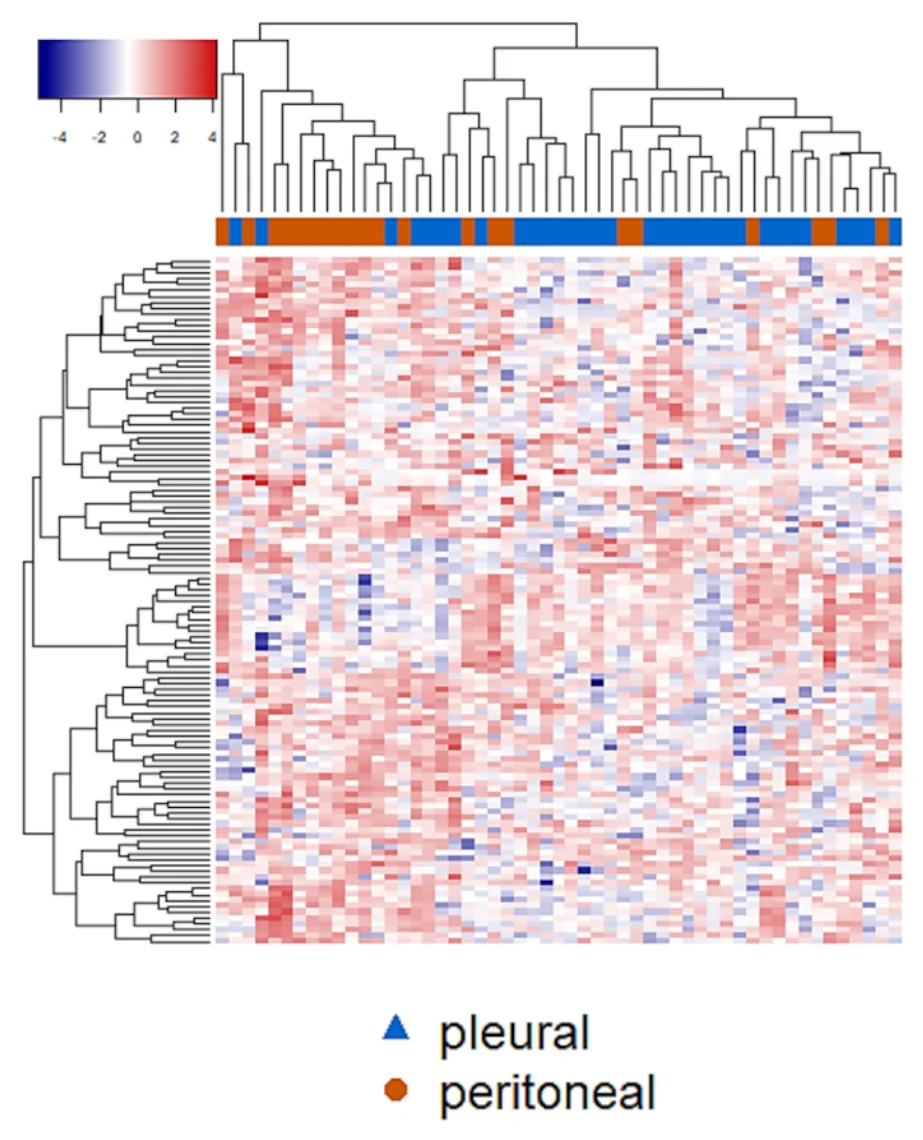
Heatmap of the mRNA-normalized data, scaled to give all genes equal variance and generated via unsupervised clustering. The orange color indicates PL, while light blue indicates PE. In addition, red indicates high expression, while blue denotes low expression, as indicated in the legend.

The housekeeping genes used for data normalization showed stable expression across all analyzed samples, confirming their suitability as internal controls. No samples were excluded following the normalization process. Overall, PL and PE showed partially overlapping gene expression profiles, although two partially separable clusters could be identified. Figure 1 shows the heatmap of the normalized mRNA expression data generated using an unsupervised hierarchical clustering approach.

**Figure 2:**
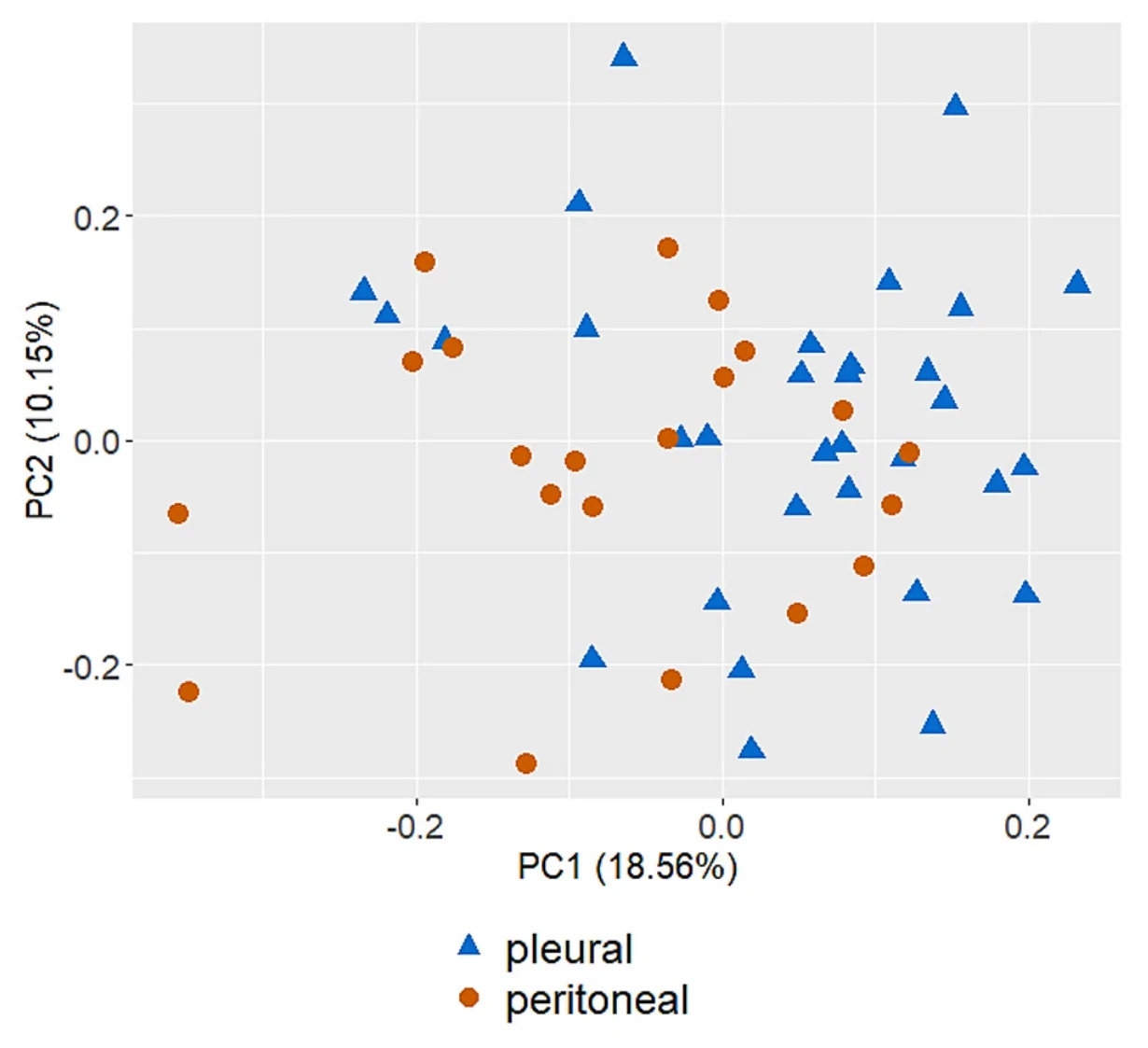
PCA of normalized gene expression data in PL and PE samples. Samples are plotted along the first two principal components (PC1 and PC2), which account for the highest proportion of variance in the dataset. Each point represents an individual sample, and colors indicate sample groups: blue represents PE, while orange represents PL.

PCA revealed a modest separation between PL and PE samples along PC1, with some overlap between the two groups. Figure 2 shows the samples plotted according to the first two principal components, PC1 (18.56% of the variance explained) and PC2 (10.15% of the variance explained), which accounted for the major sources of variability in the dataset.

### 3.3. Differential Gene Expression Analysis

**Figure 3:**
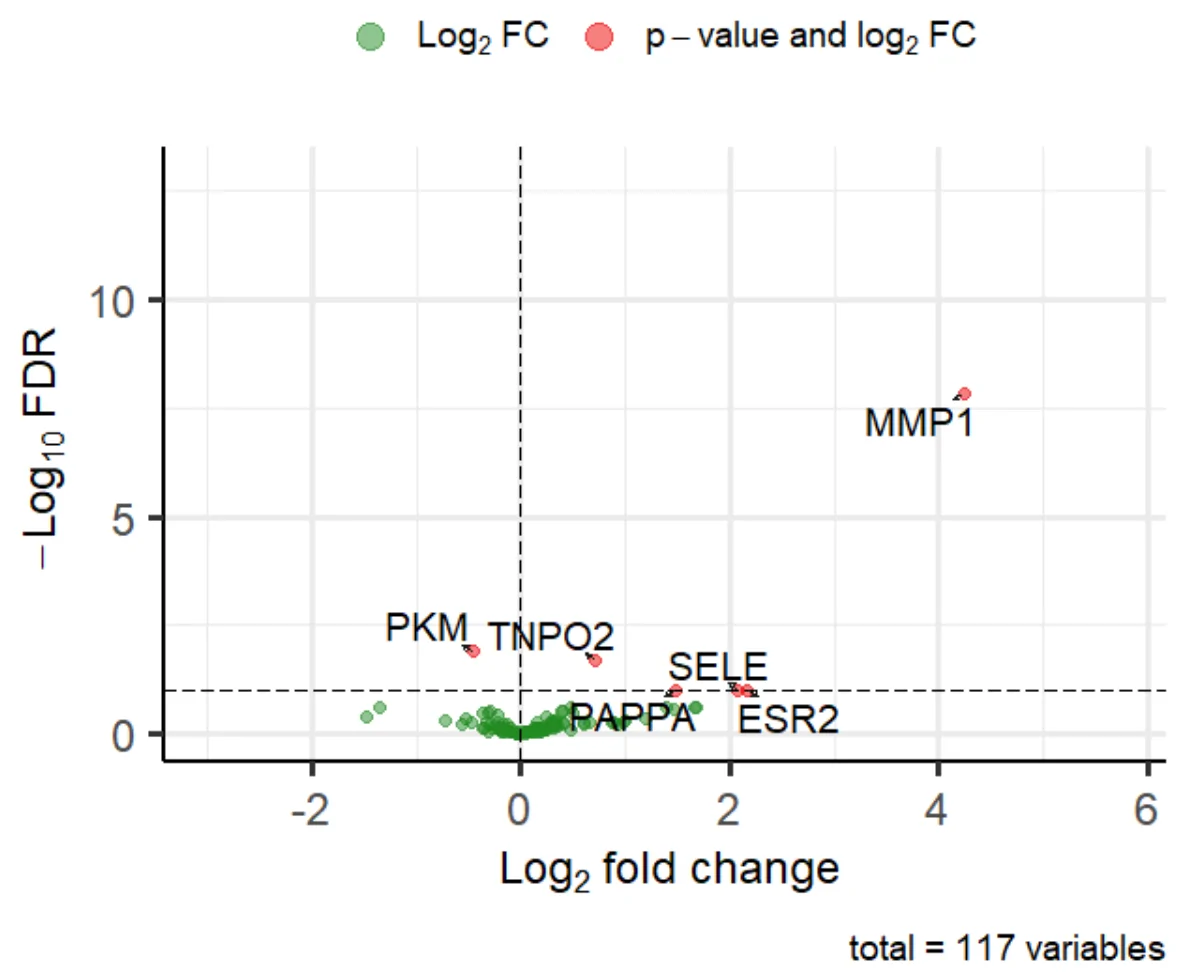
Volcano plots displaying −log10 FDR on the y-axis and log2 fold-change on the x-axis. Highly statistically significant genes fall at the top of the plot. The horizontal line indicates the FDR threshold of 0.1. Genes are red if the resulting FDR is below the given FDR threshold; otherwise, they are green, as we can see in the legend above the figure.

A comparative gene expression analysis was performed between PE and PL samples. As shown in the volcano plot in Figure 3, comparison of PE with PL revealed a significant differential expression of six genes. Among these, pyruvate kinase M1/2 (*PKM*) was significantly downregulated, whereas matrix metalloproteinase-1 (*MMP1*), E-selectin (*SELE*), estrogen receptor-*2* (*ESR2*), transportin-2 (*TNPO2*), and pappalysin-1 (*PAPPA*) were significantly upregulated in PE in comparison to PL. The complete lists of deregulated genes are reported in Table S1A. To account for potential confounding effects of age and sex, the PL–PE differential expression analysis was repeated using a model including histology, age, and sex as covariates. All six genes identified in the unadjusted analysis retained the same direction of effect in the adjusted model (Table S1B). *MMP1* remained markedly upregulated in PE relative to PL, while *TNPO2* and *PAPPA* were also upregulated. *PKM* remained significantly downregulated in PE. ESR2 and *SELE* retained positive effect estimates in PE but no longer met the predefined FDR threshold of 0.10 after adjustment, consistent with the reduced statistical power resulting from covariate adjustment in this small cohort.

**Figure 4:**
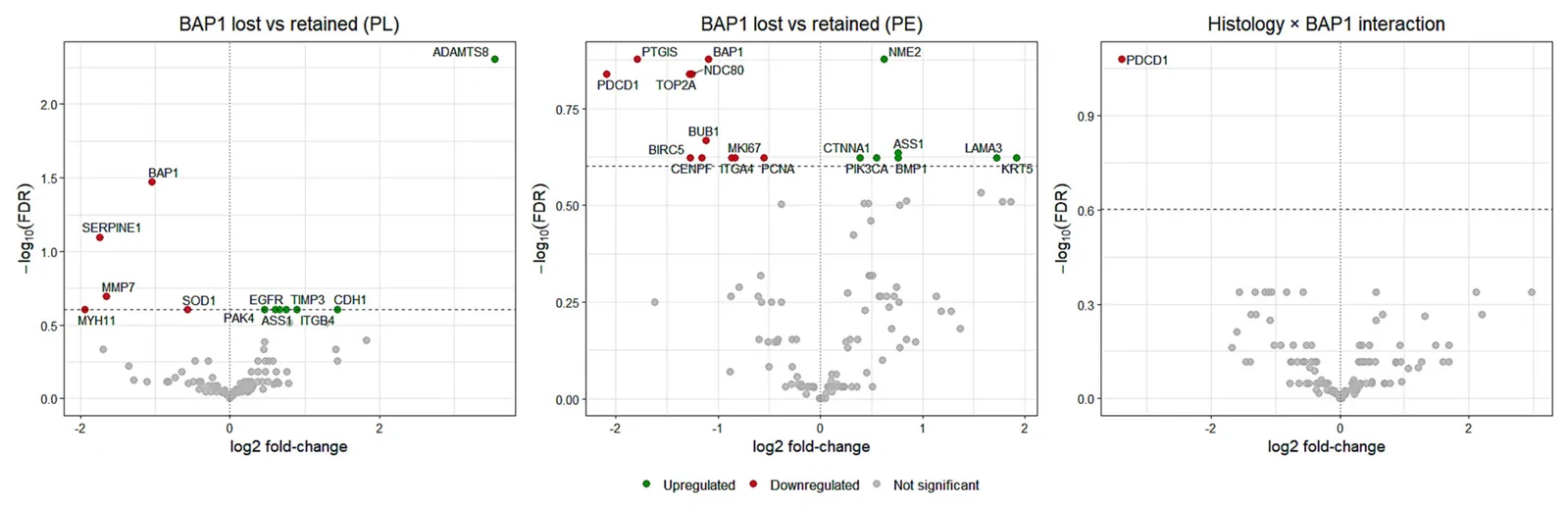
Combined volcano plots of BAP1-associated transcriptional changes in PL and PE mesothelioma. Volcano plots depict log2 fold-change (BAP1-lost vs. BAP1-retained) on the x-axis and the negative log10-transformed adjusted *p*-value on the y-axis. Differentially expressed genes at an exploratory FDR threshold of 0.25 are highlighted, with upregulated and downregulated genes shown in green and red respectively.

To explore the transcriptional correlates of BAP1 protein status, we performed a differential expression analysis stratified by BAP1 IHC (lost vs. retained), modeling histologic site (PL vs. PE) and its interaction with BAP1. The four subgroups obtained were PL with BAP1 retained (*n* = 12), PL with BAP1 lost (*n* = 19), PE with BAP1 retained (*n* = 11), and PE with BAP1 lost (*n* = 9). Using an exploratory FDR threshold of 0.25, BAP1 loss in PL was associated with deregulation of several genes, including downregulation of BAP1 itself at the mRNA level, in line with the IHC results, and altered expression of ADAMTS8 (Figure 4, left panel; Table S2). In PE, BAP1 loss was instead associated with reduced expression of *PTGIS*, *PDCD1* and cell-cycle-related genes such as *TOP2A* and *NDC80*, and with increased expression of *NME2* (Figure 4, middle panel; Table S2). Genes with a potentially different BAP1-associated effect between PL and PE were identified by the histology × BAP1 interaction term, which highlighted *PDCD1* as numerically higher in BAP1-retained than in BAP1-lost tumors (Figure 4, right panel; Table S2). This subgroup-specific finding should be interpreted cautiously given the small sample size within each subgroup.

**Figure 5:**
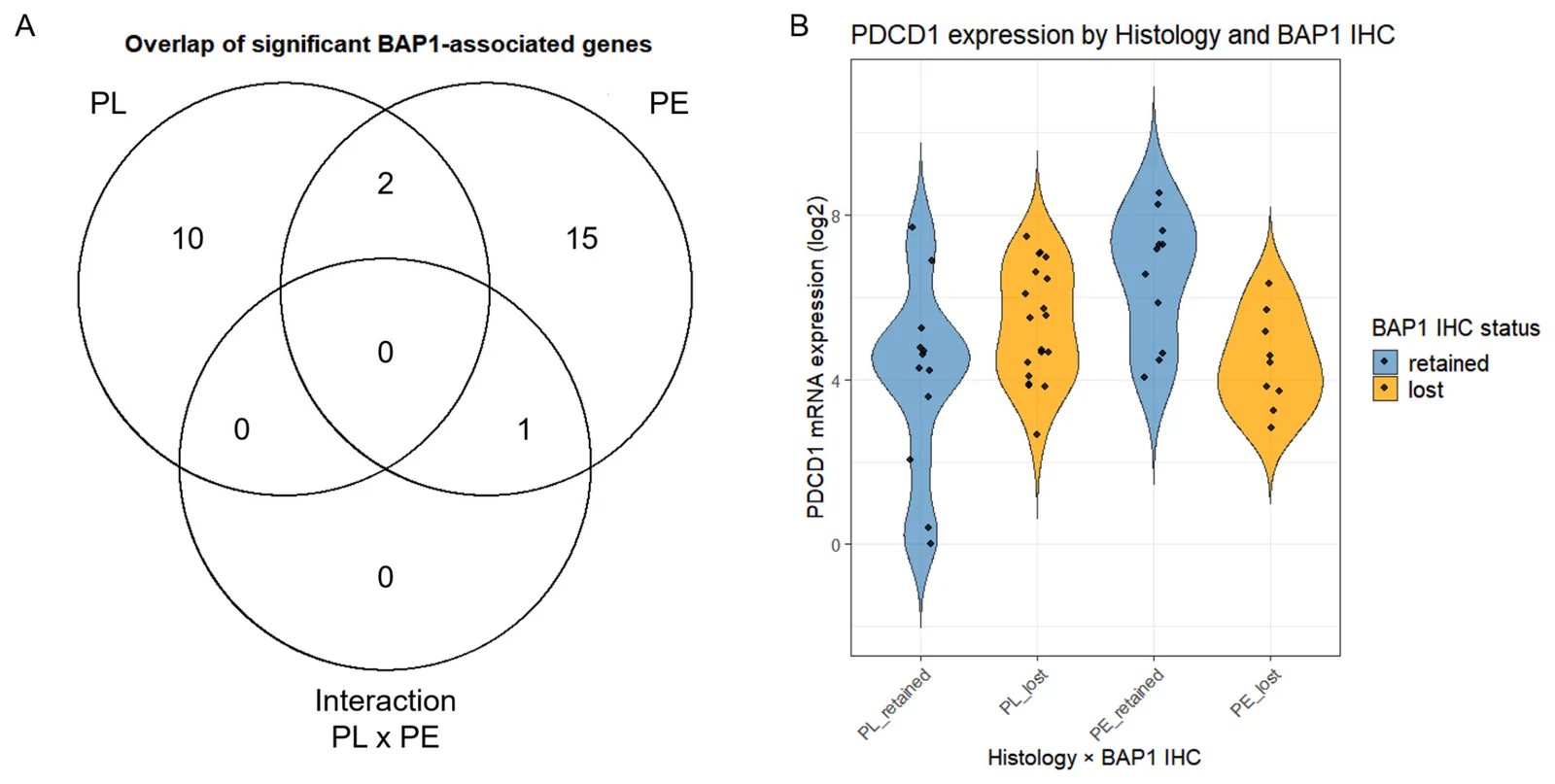
Overlap of BAP1-associated genes in pleural and peritoneal mesothelioma and BAP1-dependent modulation of *PDCD1* expression. (**A**) Venn diagram summarizing genes associated with BAP1 protein status in PL and PE mesothelioma, together with those identified by the histology × BAP1 interaction term, at an exploratory FDR threshold of 0.25. The diagram highlights both site-specific and shared BAP1-associated transcripts, including BAP1 itself and ASS1, which showed increased expression associated with BAP1 loss in both PL and PE. (**B**) Violin plot showing *PDCD1* mRNA expression across the four histology/BAP1 IHC groups (PL-retained, *n* = 12; PL-lost, *n* = 19; PE-retained, *n* = 11; PE-lost, *n* = 9), illustrating a modest increase in *PDCD1* levels in PL with BAP1 loss and a more pronounced upregulation in PE with BAP1-retained compared with BAP1-lost PE.

The overlap and specificity of BAP1-associated genes in PL and PE, as well as those identified by the interaction analysis, are summarized in the Venn diagram shown in Figure 5A. Among the genes shared between PL and PE, BAP1 itself was consistently downregulated in BAP1-lost cases, as expected, whereas *ASS1* showed increased expression associated with BAP1 loss in both sites (Figure 5A; Table S2). Finally, *PDCD1* mRNA levels, selected as a representative BAP1- and interaction-dependent gene, were visualized using a violin plot across the four histology/BAP1 IHC groups (PL-retained, PL-lost, PE-retained, PE-lost), showing a modest increase in *PDCD1* expression in PL with BAP1 loss and, more notably, higher *PDCD1* levels in PE with BAP1 retained compared with BAP1-lost PE (Figure 5B).

### 3.4. Ki67-Related Transcriptional Programs in Peritoneal Mesothelioma

**Figure 6:**
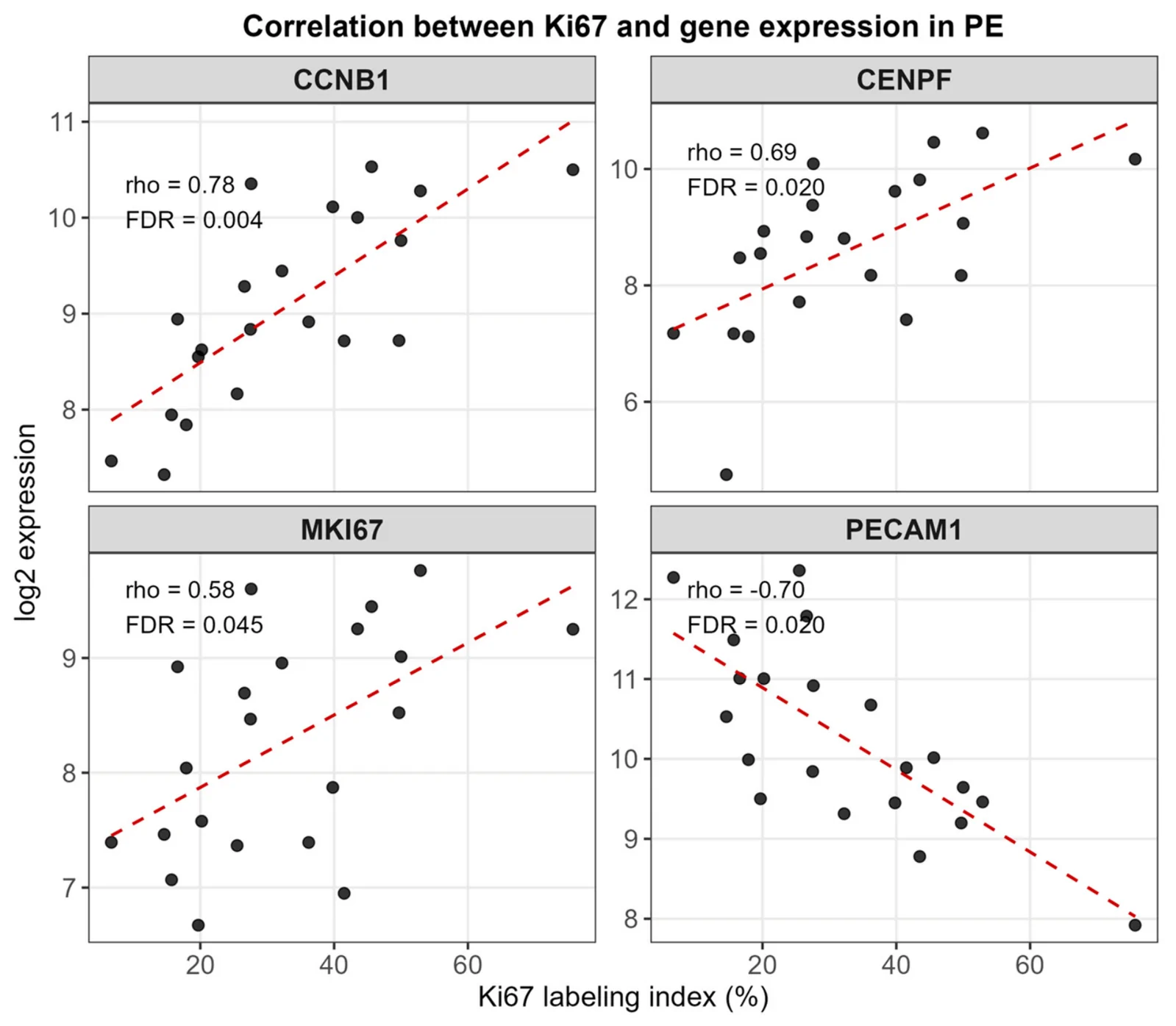
Correlation between Ki67 labeling index and gene expression in peritoneal mesothelioma. Scatter plots show the relationship between Ki67 labeling index (%) and log2 mRNA expression of *CCNB1*, *CENPF*, *MKI67* and *PECAM1* across PE samples, with a dashed linear regression line overlaid for each gene. Spearman correlation coefficients (rho) and FDR-adjusted *p*-values are reported within each panel, illustrating strong positive Ki67–gene expression correlations for cell-cycle-related transcripts (*CCNB1*, *CENPF*, *MKI67*) and an inverse correlation for the endothelial marker *PECAM1* in PE.

In PE, Ki67 has been reported as a prognostic marker and surrogate of proliferative activity. In an exploratory analysis restricted to PE, the Ki67 labeling index showed strong positive correlations with several cell-cycle-related genes, including *CCNB1*, *CENPF*, *BIRC5*, *TOP2A*, *BUB1*, *CCNB2*, *PLK1* and *MKI67* (Spearman ρ ≈ 0.58–0.78; FDR ≤ 0.05), in line with its role as a proliferation marker. *MKI67*, the gene encoding the Ki67 protein, displayed a robust association with the Ki67 index (ρ ≈ 0.58; FDR ≈ 0.045), providing an internal consistency check between transcriptomic and immunohistochemical readouts. In contrast, the BAP1-associated transcripts of primary interest, including *PDCD1*, *ASS1*, *BAP1*, *PTGIS* and *NME2*, did not show significant correlations with Ki67, indicating that their modulation in PE is unlikely to be solely explained by differences in proliferative activity. The complete correlation results are provided in Table S3. To illustrate these associations, the three genes showing the strongest positive or negative correlations (*CCNB1*, *CENPF* and *PECAM1*), together with *MKI67* as a control, were plotted against Ki67 labeling index in PE (Figure 6).

## 4. Discussion

Mesothelioma is a rare, highly aggressive malignancy associated with asbestos exposure and originating from serosal membranes. While PL, the most common form, has been extensively characterized at the molecular level, relatively little is known about the biology of PE.

In this study, we compared the gene expression profiles of 32 epithelioid PL and 21 epithelioid PE cases using a custom nCounter panel targeting 117 mesothelioma-related genes [18]. Only the epithelioid histotype was included in this study because it is the most common histological subtype.

Our findings revealed a moderate separation of gene expression profiles with some overlapping for PE and PL. We identified six differentially expressed genes between these two entities, with PE showing a deregulation of genes involved in extracellular matrix remodeling, collagen organization, and NF-κB-driven inflammatory and angiogenic pathways. Specifically, compared with PL, PE showed downregulation of *PKM* and upregulation of *MMP1*, *SELE*, *PAPPA*, *ESR2*, and *TNPO2*.

Pyruvate kinase (*PKM*) is a key regulator of the Warburg effect, whereby cancer cells preferentially rely on aerobic glycolysis instead of oxidative phosphorylation to meet their metabolic demands [20]. Matrix metalloproteinases (MMPs) are key enzymes involved in the degradation of the extracellular matrix, which play critical roles in tumor invasion and metastatic progression across a wide range of malignancies [21].

In the study by Schelch et al., inhibition of MMP1 was found to influence the morphological and behavioral plasticity of mesothelioma cells through modulation of the MAP kinase pathway [22].

Pregnancy-associated plasma protein A (PAPPA) is a metalloproteinase that regulates insulin-like growth factor; it is associated with cholangiocarcinoma, thyroid cancer, bladder cancer, cervical cancer, squamous cell lung cancer, mesothelioma, pancreatic cancer and gastric cancer [23]. Huang et al. showed a correlation between *PAPPA* expression level and PL cell migration, highlighting the need for further investigation [24]. E-selectin (*SELE*) is being studied in the context of tumor cell extravasation, a process that involves the rolling and adhesion of tumor cells to the vascular wall [25]. Estrogen receptor-2 (*ESR2*), which encodes estrogen receptor beta (*ERβ*), has been investigated in mesothelioma for its potential role in reducing mitochondrial ATP production, increasing dependence on glycolysis, and impairing cell proliferation [26]. Transportin 2 (TNPO2) is a nuclear accessory protein related to apoptosis regulation, which is highly expressed in PL tissues and cells [27,28].

Collectively, these genes are involved in biological processes related to ECM remodeling, inflammation, angiogenesis, and cellular metabolism. As reported by Hai Hu et al., ECM remodeling and fibroblast activation in mesothelioma are implicated in tumor stiffening, angiogenesis, and immune evasion. Although these processes have been extensively investigated in PL, their role in PE remains poorly characterized [29]. Lazzari et al. evaluated factors involved in peritoneal dissemination of mesothelioma. In detail, they analyzed cancer stem cells by functional assays in primary tumor cultures derived from peritoneal mesothelioma and pseudomyxoma peritonei and evaluated the expression of relevant epithelial–mesenchymal plasticity genes to determine the interconnection between stemness and epithelial mesenchymal transition. They found that stemness and hybrid epithelial–mesenchymal states may regulate neoplastic dissemination in the peritoneum in biologically distinct clinical entities and that hybrid state analyses could improve risk stratification of PE patients undergoing cytoreductive surgery and hyperthermic intraperitoneal chemotherapy [30].

BAP1 is inactivated in approximately 60% of pleural and 70% of peritoneal mesotheliomas, with a significantly higher prevalence in epithelioid histotypes [31]. This inactivation is driven by copy number loss, truncating fusion events, as well as missense, truncating, and splice-site mutations, and BAP1 loss is commonly evaluated on tumor tissue by IHC. Here we evaluated how BAP1 loss impacts expression levels of genes involved in crucial cancer pathways and how this association varies between PL and PE. In both histotypes, BAP1 loss was consistently associated with reduced BAP1 mRNA expression, supporting the concordance between the immunohistochemical and transcriptomic findings. In BAP1-deficient PL, deregulation of *ADAMTS8* was observed. *ADAMTS8* encodes a metalloproteinase whose dysregulation has been implicated in carcinogenesis through its involvement in the degradation of extracellular matrix components and adhesion molecules, as well as the activation of growth factors [32]. In addition, it has emerged as a potential tumor suppressor in several cancer types, where it is frequently downregulated [33]. Our findings suggest that *ADAMTS8* may also be associated with the transcriptional consequences of BAP1 loss in PL, although this observation requires validation in larger cohorts.

In PE, BAP1 loss was associated with reduced expression of genes encoding proteins involved in cell-cycle regulation, including *TOP2A* and *NDC80*, as well as genes related to immune and inflammatory processes, including *PDCD1*, which encodes PD-1, and *PTGIS*. In addition, BAP1 loss in PE was associated with increased expression of *NME2*, a metastasis suppressor that has been reported to inhibit cell motility and invasion [34]. *NME2* is characteristically expressed in locally invasive, non-metastatic mesotheliomas but is downregulated in more advanced metastatic phenotypes [10]. These findings raise the possibility that BAP1 status may be associated with distinct transcriptional programs in PE; however, the biological significance of these associations remains to be established.

Interestingly, in both PL and PE, BAP1 loss is associated with *ASS1* upregulation. While sarcomatoid and biphasic histotypes are usually described as ASS1-lacking tumors [35], in epithelioid mesothelioma, its upregulation or downregulation is more controversial [28]. Barnett et al. reported a correlation between BAP1 loss and *ASS1* upregulation; moreover, they observed improved survival among patients with BAP1-negative/ASS1-expressing epithelioid PL [36]. Here, we observed the same correlation between BAP1 loss and *ASS1* upregulation also in epithelioid PE. *ASS1* surely deserves further investigation for its clinical significance in both PL and PE, for its involvement in chemoresistance and also because, in agreement with its biological role, alterations in arginine metabolism may be interesting therapeutic targets [35]. Indeed, a recent study on a cell line suggested that BAP1 loss, enhancing *ASS1* expression, may increase sensitivity to ASS1-targeted inhibition [36]. In addition, evidence from the randomized ATOMIC-Meso trial in non-epithelioid PE highlights the efficacy of arginine deprivation; a pegargiminase-with-chemotherapy combination extended overall survival beyond standard care, exhibiting a well-tolerated safety profile [37].

Immunotherapy has recently improved the therapeutic landscape of biphasic and sarcomatoid pleural mesothelioma, whereas in epithelioid histology its efficacy is reduced. It has not yet been fully elucidated whether common genomic alterations have an impact on the response to immunotherapy. Available evidence suggests that BAP1-loss is usually associated with an inflamed and immunologically active tumor microenvironment [38]; however, the link between BAP1 status and immunotherapy response is still unknown. Shrestha et al. found that BAP1-altered mesothelioma shows higher levels of immune checkpoint receptors (PD-1, PD-L1, and CTLA4) with higher cytokine secretion and an increased recruitment of T lymphocytes [39]. In contrast, Dagogo-Jack et al. in a recent study reported that the composition of the immune infiltrate was similar between BAP1-altered and BAP1 wild-type tumors [40].

Notably, *PDCD1* expression showed an exploratory histology-dependent association with BAP1 IHC status. In PE, *PDCD1* mRNA expression was numerically higher in BAP1-retained than in BAP1-lost tumors, whereas in PL only a modest increase was observed in BAP1-lost compared with BAP1-retained tumors. However, these subgroup-specific observations should be interpreted cautiously, as the analysis was based on a small and unbalanced cohort. Therefore, these findings are hypothesis-generating and require validation in independent, adequately powered cohorts.

Finally, in PE, the Ki67 labeling index showed strong positive correlations with several cell-cycle-related genes, as expected. Ki67 has been reported as a prognostic marker in PE, with higher expression associated with poorer prognosis [41]. In our cohort, the Ki67 labeling index showed a strong positive correlation with *CCNB1* and *CENPF* and an inverse correlation with *PECAM1*. CCNB1 belongs to the cyclin family and plays a key role in G2/M progression and the metaphase–anaphase transition, whereas *CENPF* regulates centromere dynamics and is overexpressed in several cancer types [42]. The overexpression of both *CCNB1* and *CENPF*, as well as Ki67, has been associated with a poor prognosis in different cancer types, such as lung cancer [42] and mesothelioma [43].

The inverse correlation between Ki67 and *PECAM1*, a cell adhesion and endothelial marker, may reflect differences in tumor composition, microenvironmental features, or vascularization. *PECAM1* is primarily expressed by endothelial cells lining blood vessels [44]. When mesothelioma cells proliferate rapidly, the tumor volume can exceed functional angiogenesis, thus resulting in a high ratio of tumor cells to mature vascular endothelium. This inverse profile between Ki67 and *PECAM1* may define a more aggressive, poorly vascularized, and hypoxic mesothelioma phenotype, which can correlate with shorter overall survival and increased resistance to conventional therapies [45,46]. Importantly, the BAP1-associated transcripts of primary interest did not show significant correlations with Ki67, suggesting that their modulation in PE is unlikely to be explained solely by differences in proliferative activity.

Several limitations of this study should be acknowledged. First, the sample size was limited because of the rarity of this disease and the monocentric nature of the study. This limited statistical power, particularly for subgroup and multivariable analyses. Although age and sex were comparable between groups in our cohort and adjustment for these variables preserved the direction of effect for all six histology-associated genes, information on other potentially relevant factors, including asbestos exposure, disease stage, and treatment history, was absent or insufficiently complete for robust multivariable adjustment. Therefore, the observed transcriptional differences should be interpreted as associations with histological site rather than as definitive intrinsic effects of anatomical location.

By targeting only 117 genes, the nCounter panel does not allow for a comprehensive characterization of the transcriptomic landscape, potentially omitting key site-specific transcripts outside the preselected panel. Although manual macrodissection was used to enrich for tumor tissue prior to RNA purification, non-tumor cell contamination cannot be entirely excluded. Transcripts for genes such as *PDCD1* and *PECAM1* may also originate from tumor-infiltrating lymphocytes and vascular endothelial cells, respectively. Therefore, validation using complementary techniques, such as multiplex immunofluorescence, is required to properly interpret these preliminary findings. According to our institutional diagnostic procedures, Ki67 data were available only for PE cases, where it is routinely tested. In PL cases, Ki67 data were only partially available, and according to the quantity and quality of retrospective tissues, it was not possible to perform the analysis on all cases; for this reason, PL cases were excluded from statistical analysis. Future studies should investigate whether the associations between Ki67 and gene expression observed in PE are also present in PL.

Overall, although preliminary and hypothesis-generating, our study provides new insights into the molecular differences between epithelioid PL and PE. The identification of dysregulated genes and biological pathways, together with their potential interrelationships, may contribute to a better understanding of the distinct biological features of these rare tumors and may support the development of novel therapeutic strategies. This is particularly relevant for PE, whose management remains challenging because of its rarity, biological heterogeneity, and limited clinical evidence. Future research in independent cohorts should validate the identified transcriptional alterations and investigate their association with tumor biology, disease progression, prognosis, and treatment response.

## 5. Conclusions

In this study, we demonstrate that epithelioid PL and PE exhibit partially overlapping gene expression profiles. However, compared to epithelioid PL, epithelioid PE showed a significant upregulation of *MMP1*, *SELE*, *PAPPA*, *ESR2*, and *TNPO2*, alongside a downregulation of *PKM*. These deregulated genes are involved in relevant biological processes, including extracellular matrix remodeling, tumor cell migration, angiogenesis, inflammation, and metabolic regulation, and therefore warrant further investigation as potential epithelioid PE-specific biomarkers.

Furthermore, analysis of the relationship between BAP1 loss and gene expression revealed both shared and site-specific transcriptional features between epithelioid PL and PE, including the consistent upregulation of *ASS1* following BAP1 loss in both sites. Given that BAP1 loss is one of the most frequent molecular alterations in mesothelioma, these findings warrant further investigation to clarify its biological and potential clinical significance and to refine its role as a diagnostic, prognostic, or predictive biomarker. Larger, independent cohorts integrating molecular, pathological, and clinical data will be required to validate these findings and determine whether the identified alterations may ultimately contribute to more precise site-specific biomarker development and therapeutic strategies.

## Figures and Tables

**Table 1:** Study cohorts.

Clinical-Pathological Features			PL (*n* = 32)	PE (*n* = 21)	*p*-Value
Age (years)	median (IQR)		70 (64.75–74)	71 (59–77)	0.70
Sex	male	*n* (%)	23 (72%)	18 (86%)	0.32
	female	*n* (%)	9 (28%)	3 (14%)	
Specimen type	surgical	*n* (%)	20 (63%)	10 (48%)	0.25
	biopsy	*n* (%)	12 (27%)	11 (52%)	
Grade	low	*n* (%)	19 (60%)	14 (67%)	1
	high	*n* (%)	11 (34%)	7 (33%)	
	NA *	*n* (%)	2 (6%)	0	
BAP1	lost	*n* (%)	20 (63%)	9 (43%)	0.26
	retained	*n* (%)	12 (37.5%)	11 (52%)	
	NA *	*n* (%)	0	1 (5%)	
Asbestos exposure	yes	*n* (%)	23 (72%)	0	na
	no	*n* (%)	3 (9%)	0	
	NA *	*n* (%)	6 (19%)	21 (100%)	
Ki67 (%)	median (IQR)		NA	27.6 (19.7–43.5)	na

PL, pleural mesothelioma; PE, peritoneal mesothelioma; IQR, interquartile range; NA, not available; na, not applicable. * NA cases were not considered for statistics.

## Data Availability

Data supporting the findings of this study are available from the authors on reasonable request.

## Supplementary Materials

The following supporting information can be downloaded at: https://www.mdpi.com/article/10.3390/cancers18183018/s1, Table S1: List of deregulated genes (A) and deregulated genes adjusted by age and sex (B); Table S2: BAP1 lost vs. retained in PL (A); BAP1 lost vs. retained in PE (B); Histology × BAP1 interaction (C); Table S3: Correlation between Ki67 and gene expression.

## Abbreviations

The following abbreviations are used in this manuscript:

PL	Pleural mesothelioma
PE	Peritoneal mesothelioma
PCA	Principal component analysis
FFPE	Formalin-fixed paraffin-embedded
ECM	Extracellular matrix modification
BAP1	BRCA1-associated protein 1
DEGs	Differentially expressed genes
FDR	False discovery rate
IHC	Immunohistochemistry
